# Immunotherapy – 2068. Immunomodulatory and safety profile of a novel anti-allergic vaccine based on allergens from dermatophagoides siboney and a combination adjuvant, in sensitized mice

**DOI:** 10.1186/1939-4551-6-S1-P151

**Published:** 2013-04-23

**Authors:** Wendy Ramírez González

**Affiliations:** 1 Allergens Laboratory, National Center of Bioproducts, Mayabeque, Cuba

## Background

Th1/Tr1 promoting adjuvants, in allergen-specific vaccines, could be a valuable alternative to the current allergen immunotherapy. It is now the pro-Th1 adjuvant effect of *Neisseria meningitidis* proteoliposome (PL). Previous studies in a prophylactic murine model of respiratory allergy the vaccine showed its immunogenicity and safety. To assess the immunogenicity and non-clinical safety profile of a novel anti-allergic vaccine candidate based on purified allergens from *Dermatophagoides siboney* and a combination adjuvant containing PL and Alum, in sensitized mice.

## Methods

In therapeutic experimental setting C57/Bl6 mice were first sensitized administering *D. siboney* allergen by IP route and exposing mice to allergen aerosols. Later, allergic mice were treated with 3 doses of the adjuvanted vaccine (2µg of Der s1) by subcutaneous route. Further, mice were subjected to inhalation allergen challenge. IgE, IgG1, and IgG2a allergen-specific antibody response was measured by ELISA. The specific cellular response (IL-13, IL-10 and INF-g), were evaluated in supernatants of Lymphocyte cultures stimulated with the allergen, by eBioscience kit for FACS. Systemic toxicity was assessed measuring body weight and macroscopic and histological examination of several organs and injection site.

## Results

The experimental model reproduces the allergic condition with allergen-specific IgE production; increased blood eosinophils and Histology of lung tissues show all the characteristic of allergy inflammation. As in the previously tested preventive model, the vaccine induces a strong IgG and IgG1 antibody responses significantly higher than control groups, and moderate levels of IFN-g, although together with Tr1 (IL-10) and Th2 (Il-13) cytokines. After challenge, it was noted a significant increase (p<0.05) of the IgG/IgE ratio, with a decrease in bloods eosinophils and allergic inflammation in lung tissues as compared to placebo. No significant differences between body weights and differential leukocyte count in treated mice and placebo group was observed. Normal histology was observed. Local tolerance at the injection site suggests that granulomes in vaccinated subjects are caused by alum.

## Conclusions

The adjuvanted vaccine does not exacerbate the allergic response nor promote Th1 inflammation, supporting a satisfactory safety profile for further clinical trials in humans. This immunomodulatory effect suggests clinical benefits both in cellular and blocking antibody responses.

